# Mutational spectrum of SARS-CoV-2 during the global pandemic

**DOI:** 10.1038/s12276-021-00658-z

**Published:** 2021-08-27

**Authors:** Kijong Yi, Su Yeon Kim, Thomas Bleazard, Taewoo Kim, Jeonghwan Youk, Young Seok Ju

**Affiliations:** 1 Graduate School of Medical Science and Engineering, Korea Advanced Institute of Science and Technology (KAIST), Daejeon, 34141 Korea; 2 National Institute for Biological Standards and Control, Blanche Lane, South Mimms, Potters Bar, Hertfordshire, EN6 3QG UK; 3 GENOME INSIGHT Inc, Daejeon, 34051 Korea

**Keywords:** Evolutionary biology, Genome evolution

## Abstract

Viruses accumulate mutations under the influence of natural selection and host–virus interactions. Through a systematic comparison of 351,525 full viral genome sequences collected during the recent COVID-19 pandemic, we reveal the spectrum of SARS-CoV-2 mutations. Unlike those of other viruses, the mutational spectrum of SARS-CoV-2 exhibits extreme asymmetry, with a much higher rate of C>U than U>C substitutions, as well as a higher rate of G>U than U>G substitutions. This suggests directional genome sequence evolution during transmission. The substantial asymmetry and directionality of the mutational spectrum enable pseudotemporal tracing of SARS-CoV-2 without prior information about the root sequence, collection time, and sampling region. This shows that the viral genome sequences collected in Asia are similar to the original genome sequence. Adjusted estimation of the d*N*/d*S* ratio accounting for the asymmetrical mutational spectrum also shows evidence of negative selection on viral genes, consistent with previous reports. Our findings provide deep insights into the mutational processes in SARS-CoV-2 viral infection and advance the understanding of the history and future evolution of the virus.

## SARS-CoV-2: Monitoring coronavirus mutations

Sequencing the genetic material of >350,000 samples of the SARS-CoV-2 virus responsible for the COVID-19 pandemic has revealed details of the spectrum of mutations occurring in the recent viral transmission. The analysis was performed by researchers in South Korea and the UK, led by Young Seok Ju at the Korea Advanced Institute for Science and Technology, Daejeon. The results confirm that mutations in the SARS-CoV-2 genome are being shaped in a specific pattern: the nucleic acid bases cytosine and guanine have been replaced by uracil much more often than its reverse. Overall, the genome diversity of SARS-CoV-2 has not yet reached equilibrium, suggesting that the virus entered the human population very recently and is currently evolving rapidly. The findings enhance understanding of the history of the virus and help predict possibilities for its future evolution.

## Introduction

Severe acute respiratory syndrome coronavirus 2 (SARS-CoV-2) is spreading rapidly and globally^1,2^. Phylogenetically, SARS-CoV-2 belongs to the subgenus Sarbecovirus, a branch of *Betacoronavirus* in the *Coronaviridae* family^3^. Recent comparative studies proposed bats^4,5^ and pangolins^6–8^ as possible natural reservoirs of the virus.

Acquisition of new mutations in viral genomes and natural selection acting on the resultant phenotypic diversity are the two constituent processes of viral evolution^9^. Analogous to Darwinian evolution occurring in the origins of species, mutant viral strains gain a driving force when they acquire variations that increase infectivity and survival in the host environment. Therefore, understanding the genome changes of SARS-CoV-2 during the recent outbreak and their proper interpretations are critical for developing preventive, diagnostic, and therapeutic strategies against the virus. Although hundreds of thousands of SARS-CoV-2 genomes have been sequenced^10,11^, interpretations of the mutations as a whole have not been conducted.

Mutational signature analyses have been widely applied to cancer genomes to understand the mutational processes operative in human somatic cells^12–14^. Unique patterns of mutations provide deep insights into the mechanistic sources of genomic mutations active in the somatic lineages of cancer cells. These approaches also suggest environmental exposure of the cellular lineage, as seen in transmissible cancers^15^. Here, we apply similar approaches to the catalog of mutations collected from a considerable number of sequenced SARS-CoV-2 genomes (*n* = 351,525). Then, using the spectrum, we trace the origin of SARS-CoV-2 spreading in the human population and speculate on the molecular mechanisms that have shaped SARS-CoV-2 evolution in the recent outbreak and subsequent functional impacts of the mutations.

## Materials and methods

### Data collection and processing

An overview of the steps in our analysis is shown in Supplementary Fig. 1. A set of 351,525 prealigned full-length SARS-CoV-2 genome sequences were downloaded on 1/17/2021 from GISAID (Global Initiative On Sharing All Influenza Data^10^) (Supplementary Table 1). Viral sequences in other orders, families, and genera (e.g., *Alphacoronavirus*), as well as those of several bat coronaviruses, were downloaded from GenBank^10^ (Supplementary Table 2). Seven pangolin CoV sequences were downloaded from GISAID with accession numbers EPI_ISL_410538 to EPI_ISL_410544.

The sequences of the viruses other than SARS-CoV-2 were aligned using Kalign 3.2.3^10^. The phylogenetic trees were constructed using FastTree 2.1.11^16^, a tool for building an “approximately maximum likelihood” phylogenetic tree with default parameters. All the initial multiple sequence alignments were manually reviewed using Aliview^10^. The trees constructed were also manually reviewed using the ‘ape’ and ‘phangorn’ R packages^17,18^. With Wuhan-Hu-1 (NC_045512.2, EPI_ISL_402125)^19^ as a temporary root sequence, ancestral alleles in the internal nodes were predicted using the ‘ancestral.pml’ function with the ‘type = MPR’ option in the ‘phangorn’ R package. Mutations were called from the edges of the tree only when the mutated base could be unambiguously assigned to the conserved neighborhood, and the two upstream and two downstream bases from the mutated base had to be identical between the nodes being compared. Multiple base substitutions and short indels (<15 bases) were called in a similar way.

### Visual inspection of highly recurrent mutations

For a better understanding of the recurrent substitutions at position 11,083, we randomly selected and downloaded ~30 Illumina short-read sequencing datasets from the SRA database and reviewed them using the Integrative Genomic Viewer^20^.

### Reconstruction of the mutational spectrum

For mutational signature analysis, single-base substitutions were classified into 192 subclasses: 4 types of the original bases (4) mutated to the other bases (×3) in the context of immediate upstream (×4) and downstream (×4) bases. In the given set of substitutions, the mutational spectrum was defined by the numbers of each class of substitutions. We performed a subsampling analysis, which revealed that ~200 base substitutions are necessary to reconstruct a stable mutational spectrum (Supplementary Fig. 2). Of note, >220 K base substitutions were used for our final spectrum. Mutations on the 2nd–6th terminal branches exclusively shared among patients from the same country were defined as country-specific mutations. Terminal branch mutations were not considered for country-specific mutations because many sequencing errors are enriched on the terminal branches. The same approach was applied by annotating mutations with sampling month instead of country to investigate mutational spectrum changes depending on the virus collection month. The frequency patterns of APOBEC1 and APOBEC3 family enzyme sites were adopted from previous studies^21,22^ and represented as base frequency plots using the ‘ggseqlogo’ R package^23^.

### Maximum-likelihood estimation of the origin of SARS-CoV-2

Using the mutational spectrum of SARS-CoV-2, we estimated the root by a likelihood method, which computes the likelihood of the root by multiplying conditional probabilities of observing the descendant node base given the ancestral base across all branches in the tree (top-down approach). The conditional probability for each configuration of ancestral and descendant contexts was computed based on the substitution rate estimated from the data using the observed counts for each of the 192 contexts. The estimated substitution rates per class of substitutions were directly adopted from the observed mutational spectrum by scaling the spectral numbers to sum to 1. The likelihood of being the root of each node was calculated using the sum of the logarithmic value of substitution rates of all mutations, assuming the node is the root.

### Calculation of dN/dS using the 192-context model

Variations in the methods used to calculate dN/dS come from different assumptions regarding the expected density of neutral mutations^24,25^. We adopted the concept from previous work by Martincorena et al.^26^, an application of a 192-context model to the somatic evolution of cancer. We calculated ground neutral mutation rates for one base to another in a certain position by simply dividing the count for each substitution type by the number of corresponding contexts that appeared in the specified region. For a given region in the genome (e.g., *ORF3a*), the expected number of neutral synonymous mutations was calculated by the sum of the probabilities where a base change resulted in a silent mutation. The expected number of nonsynonymous mutations was calculated in a similar way. The dN and dS values were then calculated using the observed and expected numbers of nonsynonymous and silent mutations, respectively. To estimate the confidence interval of d*N*/d*S* for each region, bootstrapping was performed by randomly resampling the observed mutations in that region. Conventional maximum likelihood methods with different models and codon frequencies were tried to evaluate natural selection using IQ-TREE software (v2.1.1)^27^.

## Results

### Detection of SARS-CoV-2 mutations

**Fig. 1:**
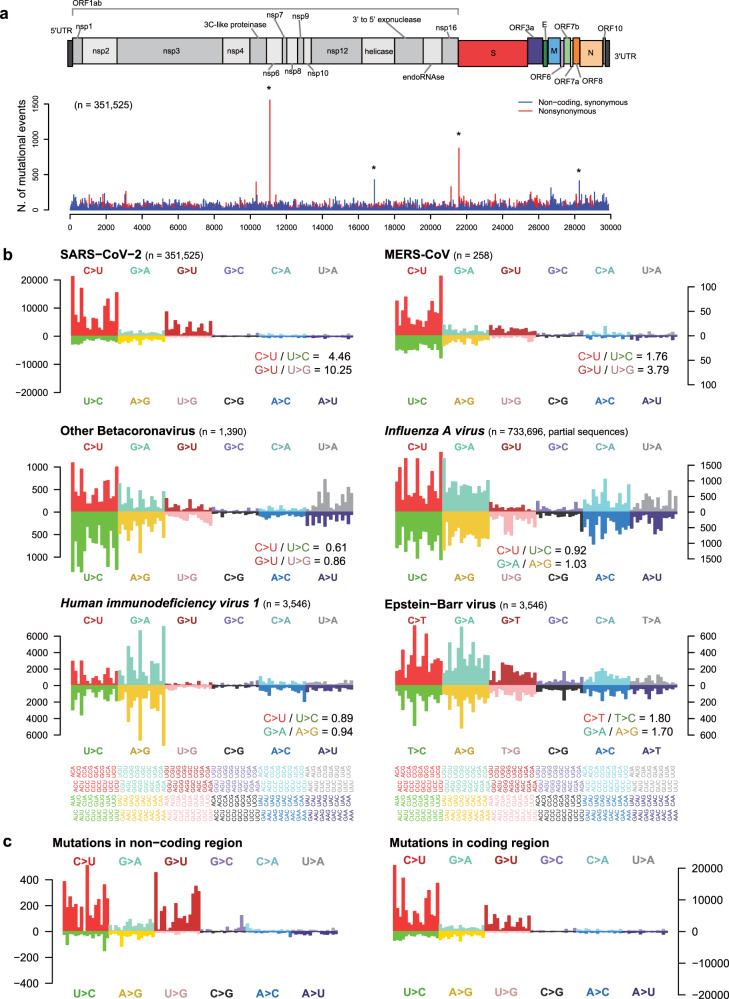
Mutational signature of SARS-CoV-2. **a** Distribution of the number of single-base substitutions along the viral genome. Each bar in the lower panel represents the counts for nonsynonymous substitutions (red) and synonymous substitutions in noncoding regions (blue). Except for a few recurrently mutated positions forming peaks, mutations are more or less uniformly distributed along the genome. The four highest peaks marked by asterisks are located in homopolymeric stretches. The highest peak at position 11,083 is caused by recurrent G→U and U→G substitutions in the context of U (5′-UUUUUUUGU-3′) (detailed in Supplementary Fig. 1). **b** Mutational signatures of SARS-CoV-2 and other viruses: MERS-CoV, other betacoronaviruses (including nonhuman hosted ones), *Influenza A virus*, HIV-1, and Epstein-Barr virus. For a given species (indicated in the panel title together with *n* = sample size), the panel shows the spectrum of observed substitution counts for 192 classes, a combination of changes of the major base (12 scenarios indicated with different colors) together with 4 types of the 5′ immediate upstream base and 4 types of the 3′ immediate downstream. Mutations of SARS-CoV-2 are particularly enriched in five sequence contexts (ACA, ACU, UCA, UCU, and GCU), and the mutational spectrum is asymmetric in terms of Watson-Crick base pairing and directional (i.e., the mutated and substituted bases are not balanced). Specifically, in SARS-CoV-2, C→U is much more frequent than U→C, and similarly, G→U is more frequent than U→G. MERS-CoV also exhibits an asymmetrical mutational spectrum similar to that of SARS-CoV-2. In contrast, *Influenza A virus* and HIV-1 show largely balanced patterns (C→U ≈ U→C and G→A ≈ A→G). Epstein-Barr virus, which is a DNA virus, shows asymmetry in its reversibility (C→T ≄ T→C) but exhibits symmetry in Watson-Crick base pairing (C→T ≈ G→A in a CpG context). **c** Comparison of mutational signatures in mutations of noncoding and coding regions.

We used 351,525 prealigned full-length SARS-CoV-2 genome sequences publicly released in the GISAID database as of January 17, 2021^10^. These sequences were collected from 82 countries from December 24, 2019, to January 12, 2021 (Supplementary Table 1). After constructing the phylogenetic tree and estimating internal node sequences (“Materials and Methods”), we compared sequences connected by each edge in the tree. We cataloged base changes, including nucleotide substitutions and short indels. From our efforts, we obtained a list of mutation sites containing 227,639 single-base substitutions, 1070 double- or triple-base substitutions, and 6001 short indels (Fig. 1a, Supplementary Fig. 3a, Supplementary Table 1, and a list of mutations with annotations in Supplementary Table 3). For comparability with other papers, we use the genomic coordinates of the conventional SARS-CoV-2 reference, NC_045512 (known as Wuhan-Hu-1), throughout the manuscript. The mutation sites were more or less uniformly distributed across the entire viral genome (Supplementary Fig. 3b).

Several sites were recurrently mutated on multiple branches, implying many independent mutational events at the same genomic sites if we neglect the small chance of viral genome recombination between different mutants. At first glance, these mutations can be interpreted as evidence of positive selection. However, with careful examination of such mutations, especially for the top four recurrent calls (Fig. 1a), these sites are recurrently mutated in both directions. For example, *ORF1ab* L3606F (11,083 G→U substitution) and its reverse F3606L (11,083 U→G substitution) occurred 1505 times and 49 times, respectively (Supplementary Fig. 4a). This implies something other than evidence of positive selection, such as either replicative or sequencing error-prone sites. Indeed, these recurrent sites were frequently located in the homopolymeric region, and even deep sequencing data of a virus pool from one patient showed reads representing both alleles with various allele frequencies (Supplementary Fig. 4b). Therefore, we speculated that these mutations might not induce obvious functional advantages in the spread of SARS-CoV-2^28^.

### Mutational signature of SARS-CoV-2

Of the 12 classes of base substitutions, the C→U transition was dominant in our mutation catalog (46.5%). Interestingly, we found substantial asymmetry in base changes. For example, the rate of the C→U transition was much higher than that of its reverse U→C substitution (46.5% vs. 9.4%, respectively). Likewise, the rate of G→U transversion was almost ten times higher than that of U→G substitution (18.2% vs. 1.3%, respectively).

To more deeply explore the mutational spectrum, we considered the sequence context of mutated bases and partitioned the 12 classes of base substitutions into 192 subclasses (i.e., 12 substitution classes × 4 types of the 5′ immediate upstream base × 4 types of the 3′ immediate downstream base; Supplementary Table 4). Under these circumstances, C→U base substitutions were further enriched in five sequence contexts, ACU, ACA, UCA, UCU, and GCU (Fig. 1b). C→U transitions in the five contexts were 7.5 times more frequent than the reverse U→C transition in the same surrounding bases. These results indicate that the proportion of uracil has increased over time, at least during the early spread of SARS-CoV-2 in the human population. Thus, the pool of viral genomes has been undergoing directional change.

Interestingly, asymmetric mutational spectra have also been found in the spread of other zoonotic betacoronavirus species, such as *Middle East respiratory syndrome coronavirus* (MERS-CoV, Fig. 1b) and SARS-CoV (11 C→U vs. 7 U→C from 11 sequences, data not shown), which are thought to have recently been introduced into the human population. Asymmetry was not found in old viruses in the human population, such as *Influenza A virus* and *Human immunodeficiency virus 1* (HIV-1) (Fig. 1b, Supplementary Fig. 5, and Supplementary Table 4).

Three factors may shape SARS-CoV-2 mutations in infected cells: (1) mistakes in viral genome replication by RNA-dependent RNA polymerase (known as RdRP), (2) active damaging processes in RNA molecules caused by host immune systems^29^, and (3) the selective advantage of possessing a certain context due to an underlying mechanism such as codon usage adaptation^30,31^. Of these, the RdRP error cannot explain the asymmetry of the mutational spectrum we observed, in particular the imbalance between substitution classes, because both positive- and negative-sense RNAs are equivalently replicated by the polymerase in infected cells (Supplementary Fig. 6).

Recently, RNA editing was suggested as a mutational process for SARS-CoV-2^29,32^. However, there are some missing links between RNA editing and our mutational spectrum. Although our mutational spectrum is quite similar to the pattern of mutations caused by APOBEC1 ([A/U]p[C→U]p[A/U]), the enzyme is exclusively expressed in the small and large intestine, which is not a predominant target organ for this viral infection. Moreover, APOBEC1-mediated RNA editing requires a mooring sequence downstream of the mutant site (WRAUYANUAU 3-10 bases downstream of the target cytosine site)^21,33^. However, in the mutations we profiled, no such sequence was found even after allowing two bases of mismatch. In addition, the mutational spectrum of APOBEC3A or APOBEC3B (Up[C→U]pN)^34,35^ was not very similar to our spectrum, although the enzymes are known to be expressed in the lung (Supplementary Fig. 7a). Indeed, asymmetry in another class of base substitutions (G→U base substitutions, which are 10.2 times more frequent than U→G changes) cannot be explained by currently known RNA-editing enzymes.

We investigated whether the asymmetry of the mutational spectrum is the outcome of selection pressure on infected human cells. Many viruses tend to have codon usage similar to that of their major host due to higher efficiency in protein translation^30,36–40^. However, the asymmetric mutational spectrum overall makes viral codon usage more dissimilar from that of humans (Supplementary Fig. 7b). Among the 20 kinds of amino acids, 15 can have uracil in the third position of the codon. When looking at all the synonymous mutations observed in SARS-CoV-2 by amino acid, in all 15 kinds, the change to codons with uracil in the third position was the most frequent. In the codons for 14 of these 15 amino acids (except arginine), the synonymous mutations most frequently produced codons that are the most used in the virus genome. Moreover, we also observed that the mutation spectrum in the noncoding region was almost identical to that in the coding region, although the mutation rate in the noncoding region was slightly higher (13.93 vs. 9.18 mutations/base in the tree of 351,525 samples) (Fig. 1c and Supplementary Fig. 7c). Overall, the mutational spectrum of SARS-CoV-2 exhibits an asymmetric, currently unknown mutational process that has been operative during transmission in the human population.

### Tracing the origin of SARS-CoV-2

Next, we used the asymmetric mutational spectrum to root the SARS-CoV-2 pandemic. Conventionally, the reference genome of SARS-CoV-2, which was sequenced from viral particles isolated from Wuhan in December 2019 (known as Wuhan-Hu-1)^19^, is considered a root in viral genome studies^41^. Additionally, previous studies used outgroup sequences, such as bat coronavirus sequences, to find the root of SARS-CoV-2^42,43^. However, due to the immoderate sequence homology among the outgroup sequences, the approaches pointed to incorrect sequences as the origin of SARS-CoV-2.

To tackle this problem, we used the directionality of the mutations to trace the root of SARS-CoV-2 without information about the sampling date. Our asymmetric mutational signature directly suggests that viral sequences harboring a higher proportion of C and G are likely to be more ancestral, that is, more similar to the original genomic sequence. To this end, we employed a likelihood approach to statistically evaluate the root from 351,525 sequences (“Materials and Methods”). We used a fixed substitution rate matrix proportional to the mutational spectrum, based on the observation that the spectrum (1) is robust to the position of the root (Supplementary Fig. 8a, b) and (2) is also uniform across the tree, regardless of the viral clades, countries of patients, or sampling dates (Supplementary Fig. 9a–c).

Our approach clearly suggested a root, which is an ancestral node of four SARS-CoV-2 sequences collected in Wuhan in the earliest period (February 2020, Fig. 2a, b; Supplementary Table 1). The maximum-likelihood node showed three U→C substitutions compared to Wuhan-Hu-1, the typical reference sequence of SARS-CoV-2. The node sequence was 440 times more probable as a root than was Wuhan-Hu-1.

**Fig. 2:**
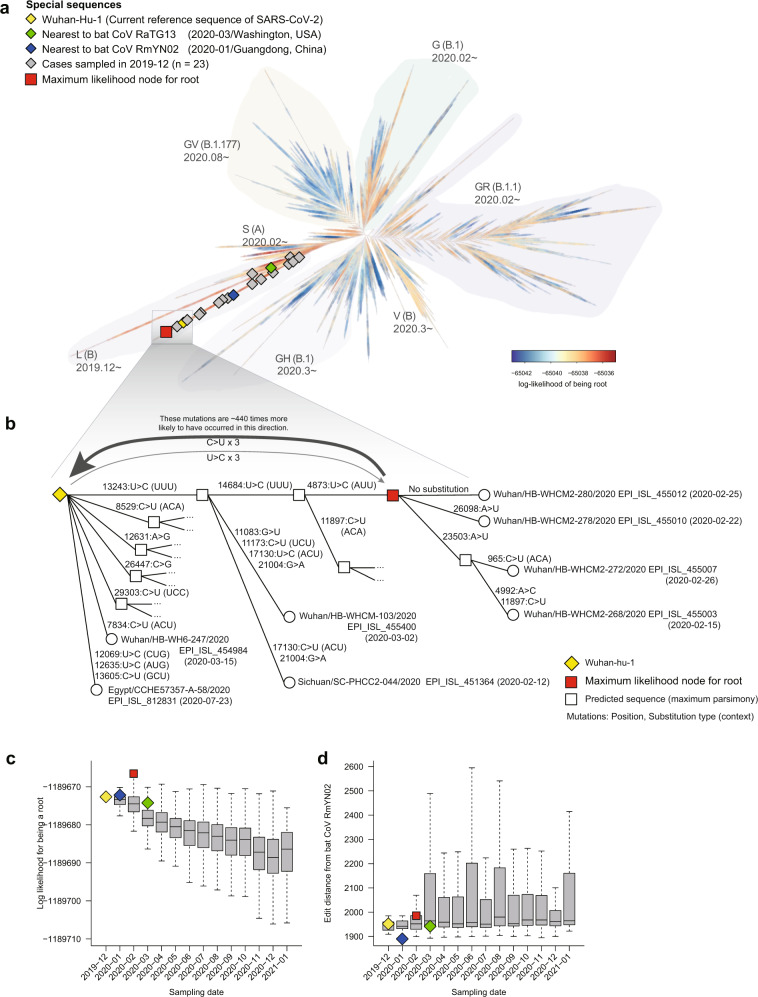
Tracing the origin of SARS-CoV-2 using its mutational signature. **a** Phylogenetic tree constructed from 351,525 full-length SARS-CoV-2 genome sequences deposited in the GISAID database. Well-known named clades are indicated with light shades of color and labeled according to widely used nomenclature^75^. Special sequences with prior knowledge: the Wuhan reference sequence (yellow), the sequence nearest to bat CoV RaTG13 (green) or RmYN02 (blue), and sequences sampled in December 2019 (gray) are marked with diamonds, and the presumed root (internal node with the maximum likelihood of being the root) is marked with a red square. The sequence data are constantly being updated on the GISAID website (https://www.gisaid.org/). **b** Detailed phylogenetic structure and associated substitutions between the presumed root (red square) and Wuhan-hu-1. **c** Boxplot showing the log-likelihood of being the root by collection time of the samples. Overall, likelihoods decrease steadily as collection time progresses. **d** Boxplot showing the edit distance from bat CoV RmYN02 by collection time of the samples. Overall, the edit distance does not significantly change over time.

The likelihood for the root of each node exhibits a trend of steadily decreasing over the collection date of the sequences, supporting the validity of our method (Fig. 2c). Such a trend was not clearly seen in the typical outgroup-based analyses (Fig. 2d). Although the sequence identity between SARS-CoV-2 and its closest bat coronavirus (RmYN02) is 96.2%, these viral genomes differ by more than 1,000 mutations. This far outnumbers the number of sequence changes between any two sequenced genomes of SARS-CoV-2 (6.4 substitutions on average). As a consequence, rooting based on the edit distance from the outgroup resulted in arbitrary outcomes^42^. In addition, the overall edit distance of the viral genomes did not significantly change over the collection dates of SARS-CoV-2 (Fig. 2d), highlighting the limitation of the outgroup approach in tracing the origin.

### Mutational signature in the natural relatives of SARS-CoV-2

**Fig. 3:**
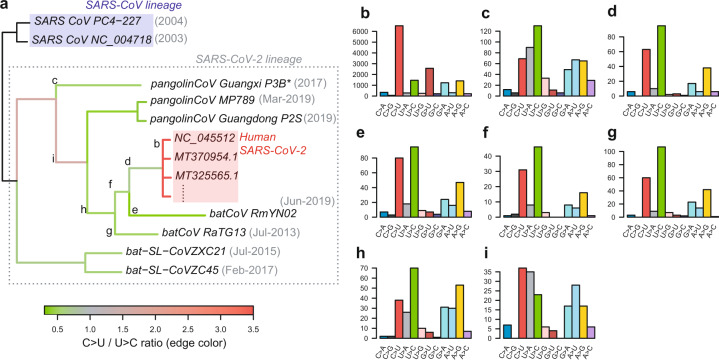
Changes in C→U preference over the course of SARS-CoV-2 divergence. **a** Phylogenetic tree of the closest natural relatives of SARS-CoV-2, which was adapted from GISAID. Substitutions between ancestral and descendant sequences were collected in the root-to-leaf direction. The substitution ratio of C→U over U→C is marked by color on each edge (reddish for more C→U and greenish for more U→C). **b**–**i** The substitution spectra for 12 major classes are shown for the spread among humans (**b**) and for each divergence lineage indicated in the middle of branches (**c**–**i**). There are more C→U than U→C changes in human SARS-CoV-2 (**b**), while the reverse pattern is shown in the divergence of bat coronaviruses (**c**–**i**). Although there is uncertainty in the mutation calls for old lineages due to a high degree of mismatch between sequences, one upper branch suggests more C→U than U→C changes (**i**). The status of the ancestral sequence requires the use of information outside the node. The pangolin coronavirus from Guanxi (asterisks) is a consensus sequence of the following six sequences from the same study: P3B, P2V, P5E, P5L, P1E, and P4L. For divergence among these sequences, mutation counts per class and the C→U/U→C ratio are not displayed because their mutation count is not sufficiently large to reliably represent them.

We investigated whether spectrum asymmetry is also present in long-term fixation in close relatives of SARS-CoV-2, such as bat coronaviruses (RaTG13 and RmYN02) and a pangolin coronavirus recently sequenced from Guangdong and Guanxi, China^4–7^. In the divergence of these viruses, the rate of U→C substitutions was higher than that of C→U substitutions (Fig. 3a–i). Similar to the spread among humans, mutations between the six pangolin coronavirus sequences from Guangxi^6^ also have more C→U substitutions than U→C substitutions, but the number of mutations is too small for confirmation (19 C→U and 9 U→C among 77 substitutions).

### Functional consequences of the SARS-CoV-2 mutations

Of the 227,639 substitutions we identified, 162,221 (58.4%) induce amino acid alterations of the SARS-CoV-2 protein-coding genes. These nonsynonymous mutations were relatively evenly distributed in proportion to the length of the gene and had various ratios of C→U transitions (Supplementary Fig. 10). A large proportion of the amino acid changes follow the preferential C→U substitution in the mutational signature. For example, the most common type of nonsynonymous mutation observed in human SARS-CoV-2 is alanine to valine (Ala→Val, *n* = 12,202; 4.6% of all nonsynonymous mutations). Of these, 7252 (59.4%) coincided with the trinucleotide context signature, occurring by GCU→GUU codon change. Similarly, of the 17,138 threonine to isoleucine (Thr→Ile, 6.4%) changes, 8124 (47.4%) occur by ACU→AUU, congruent with the mutational signature.

**Fig. 4:**
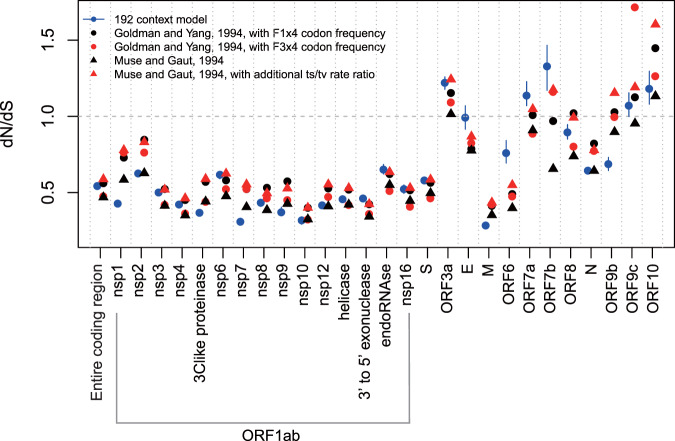
Assessment of natural selection on SARS-CoV-2 genes. **a** Across SARS-CoV-2 genes, the ratio of nonsynonymous to synonymous substitutions (dN/dS) was estimated using the 192-context model (Methods). The vertical bar indicates bootstrap variability, reflecting the size of each gene. The dN/dS ratio estimated from the entire coding region is shown as the first tick on the *x*-axis. Circles and triangles indicate the dN/dS ratio calculated based on the Goldman and Yang and the Muse and Gaut models, respectively.

The ratio of nonsynonymous to synonymous substitutions, the dN/dS ratio, is a key metric that has frequently been used for inference of natural selection^44^. Currently, the most widely used dN/dS methods are the maximum likelihood method^44^ and the counting-based method (e.g., NG86 and YN00^45,46^). These models estimate the ratio of nonsynonymous to synonymous mutations under neutral selection by estimating transition and transversion rate parameters and codon frequencies in given sequences. However, similar to base substitution, when strong asymmetry exists in the mutation spectrum, those codon substitution models may not fully capture the underlying substitutional process. The mutational signature would result in bias in the set of codon changes, and the dN/dS calculation will be skewed unless this is corrected. Here, we calculated the dN/dS ratio, taking into account both the asymmetric mutational signature and codon usage (“Methods”^47^). The adjusted calculation showed that, on average, the overall dN/dS ratio was significantly < 1, indicating that the number of nonsynonymous mutations was lower than that expected under the assumption of neutrality (Fig. 4a). Compared with the widely used Markov chain models, such as the Goldman and Yang model^48^ or the Muse and Gaut model^49^, our dN/dS method using sequence contexts generally showed similar trends, but some genes showed apparent differences (Fig. 4). The membrane protein (M) had the lowest dN/dS ratio, followed by nonstructural protein 7 (*nsp7*). Seven nonstructural genes, *ORF3a*, *ORF7a*, *ORF7b*, *ORF8*, *ORF9a, ORF9b*, and *ORF10*, by contrast, showed higher dN/dS ratios than other genes, but these values were close to one, suggesting neutral evolution. Collectively, our results indicate that the vast majority of mutations accumulated in the pool of SARS-CoV-2 genomes during transmission would be functionally disadvantageous or neutral.

## Discussion

Punctuated viral evolution is a frequently proposed concept in which a virus evolves rapidly over short periods of time followed by long periods of no change^50,51^. Such rapid evolution of new viral strains appears to contradict more clock-like evolution, and this shift is caused by a variety of factors, such as host population changes, migration, and vaccination^50–52^. The SARS-CoV-2 data contain a full spectrum of mutations that were obtained after the virus was transmitted to its new host. However, many researchers use the most widely used general time-reversible (GTR) model to build phylogenetic trees and evaluate mutation rates and selection without considering whether the evolution of SARS-CoV-2 is clock-like or punctuated^53^. Our observation clearly shows that SARS-CoV-2 was introduced to the human population very recently and is not at evolutionary equilibrium.

The C→U and G→U asymmetry in the SARS-CoV-2 and MERS-CoV mutation spectra may be a characteristic of zoonotic RNA viruses recently introduced to human tissues. Previously, linear changes in the base composition over the time of spread were observed in Ebola and influenza viruses^54^. Our results are well aligned with this observation. When zoonotic viruses invade human cells from nonhuman hosts, the human cells are not optimized to provide ideal growth conditions, and the virus base composition is not at equilibrium. In these circumstances, such directional changes in viral genome sequences are possible.

Codon composition is one of the extensively studied genomic properties of many viruses. The codon usage profile of a virus is thought to be passively formed by the mutational pressure exerted on the virus^55–57^ or to induce active translational selection itself^30,31^. Because viruses share a tRNA pool with their hosts, codon usage patterns can evolve to become similar to maximize translational efficiency^30^, or conversely, when the patterns are too similar, they can become dissimilar to avoid competition^31^. The codon usage profile of SARS-CoV-2 is known to be more similar to that of *Bungarus multicinctus* (snake) or *Rhinolophus sinicus* (bat) and not similar to that of humans, *Manis javanica* (pangolin)^58^, and *Camelus dromedarius* (camel, host of MERS-CoV)^59^. The same mutational spectrum in the noncoding region strongly implies that the mutational spectrum overall is not due to selective pressure on codon usage.

The Markov model-based unrestricted model (UNREST model^60–62^) is a possible method for estimating the root based on the asymmetry of substitutions. However, the UNREST model assumes stationary homogeneous base composition over the time of evolution. This stationarity assumption is frequently reported to be violated in empirical observations, such as continuous decay of the CpG content due to spontaneous cytosine deamination^63,64^. There are Markov-based approaches that allow a nonstationary nonhomogeneous time-irreversible nature^65,66^. However, it would be extremely difficult to apply those methods to our data (351,525 sequences) because of the requirements for large memory and long computing time. Overall, our findings demonstrate the utility of the asymmetric mutational spectrum in searching for the root sequence without imposing information about the sampling date or region^42,43^.

Our observation of U→C-dominant mutations in the evolutionary tree of SARS-CoV-2-related viruses suggests differences in the evolutionary characteristics of long-term fixation and those of accelerated (punctuated) evolution. In other words, the genetic diversity of human SARS-CoV-2 has a different mechanistic property from the high divergence of SARS-CoV-2-related viruses of natural origin. Although there is missing information between human SARS-CoV-2 and its natural relatives, making assertions difficult, the predominant C,G→U mutations in human SARS-CoV-2 infections are due to the recent host shift and act as a driving force of the punctuated equilibrium that accelerates viral evolution.

Variations from estimating the dN/dS ratios have been developed to evaluate natural selection on coding sequences. These variations include different ways to estimate nonsynonymous/synonymous mutation counts under the neutral selection assumption. This depends on the belief of how mutations occur and how four bases occupy different codon positions in a given coding sequence^67^. Here, we account for the context of mutations to estimate dN/dS. Our approach has two assumptions. One is that the observed mutation count per context class is sufficiently robust to use the crude count ratio as the actual expected mutation ratio. The other assumption is that the context-specific mutational spectrum is mainly shaped by mutational pressure but not by selection. Further studies aiming to reveal the biochemical mechanism of the C→U- and G→U-dominant mutations in SARS-CoV-2 are required to resolve these assumptions, which will require further validation in functional studies. The context-adjusted estimate of dN/dS is generally in good agreement with previous reports. For example, negative selection is observed for most genes. The diversifying selection on *ORF3* and *ORF8* is more obvious with our context-adjusted method than with conventional models^68,69^. Among the nonstructural protein groups, *Nsp7* showed the lowest dN/dS with the context-adjusted method. It seems to be under the strongest negative selection and thus could be used as a target for antiviral therapeutics^70^ or vaccine development^71–74^.

In summary, we investigated the mutational signature during the global spread of SARS-CoV-2 using 351,525 genome sequences. A highly asymmetric spectrum was observed, with an exceptionally high proportion of C→U substitutions enriched in a few sequence contexts. We speculate that SARS-CoV-2 is currently in a rapid evolutionary stage prior to reaching static equilibrium. Tracing the outbreak’s origin by utilizing the directional substitution pattern indicates that the sequences collected early in Asia are more similar to the original sequence than other sequences are. Despite many nonsynonymous mutations, we observed that the viral genomes were under negative selection.

## Supplementary information

Supplementary Information

Supplementary Tables 1-4

## Data Availability

The phylogenetic tree data are available on our website (https://github.com/ju-lab/SC2_evol_signature).
