# Supplementary material for: Mutational spectrum of SARS-CoV-2 during the global pandemic
Source: Exp Mol Med. 2021 Aug 27;53(8):1229–37. doi: 10.1038/s12276-021-00658-z (PMC8393781; doi:10.1038/s12276-021-00658-z)
Supplement: Supplementary file 1 — Supplementary Information [file 12276_2021_658_MOESM1_ESM.pdf]

Supplementary information: list and legends

Supplementary Fig. 1: Flowchart of analysis.

Supplementary Fig. 2: Estimated number of required mutations for a stable mutational signature.

Supplementary Fig. 3: Summaries of SARS-CoV-2 mutations.

Supplementary Fig. 4: *ORF1ab* L3606F mutation (base position 11,083) located in a homopolymeric sequence.

Supplementary Fig. 5: Landscape of mutational signatures in viral speciation.

Supplementary Fig. 6: Illustration of potential replication error made by RNA-dependent RNA polymerase (RdRP) in the viral genome.

Supplementary Fig. 7: Basis for characterizing the mutational spectrum of SARS-CoV-2: RNA editing, translational selection, and comparison between coding and non-coding regions.

Supplementary Fig. 8: Robustness of the SARS-CoV-2 mutational spectrum.

Supplementary Fig. 9: SARS-CoV-2 mutational spectrum conserved across times and regions

Supplementary Fig. 10: Nonsynonymous substitution counts and length-normalized counts per gene in the SARS-CoV-2 genome.

Supplementary Table 1: SARS-CoV-2 sequences used in this study

Supplementary Table 2: Other viral sequences used in this study

Supplementary Table 3: List of mutations

Supplementary Table 4: Mutational signature spectrum of SARS-CoV-2 and other viruses

Supplementary Fig. 1

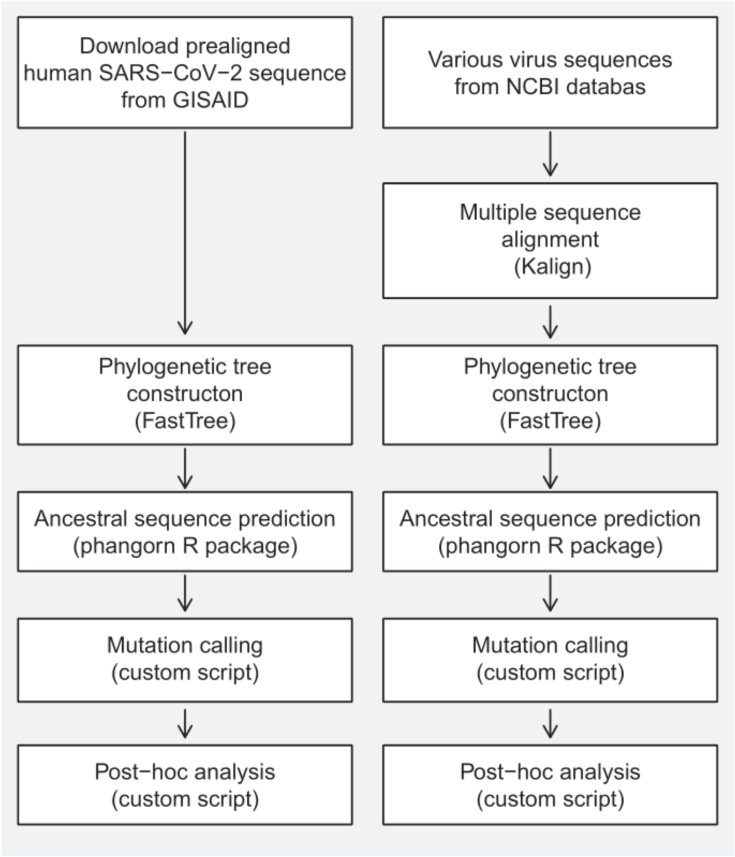

Supplementary Fig. 1: Flowchart of analysis.

Flow chart showing the analytic process in the study.

Supplementary Fig. 2

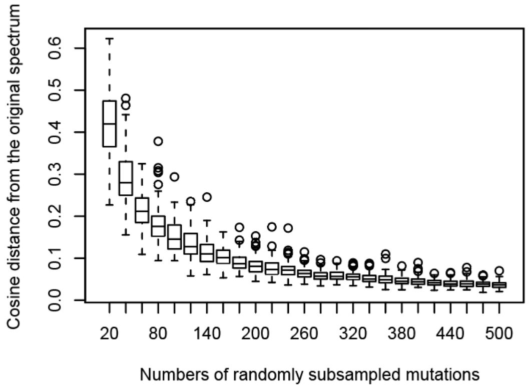

Supplementary Fig. 2: Estimating the required number of mutations for a stable mutational signature.

Distributions of cosine distances (y-axis) between the original spectrum and those from randomly subsampled mutations in different numbers (x-axis) is plotted. As the number of mutations sampled increases, it becomes similar to the original spectrum, which becomes very similar (cosine distance < 0.1) to the spectrum when the number of mutations becomes about ~200.

Supplementary Fig. 3

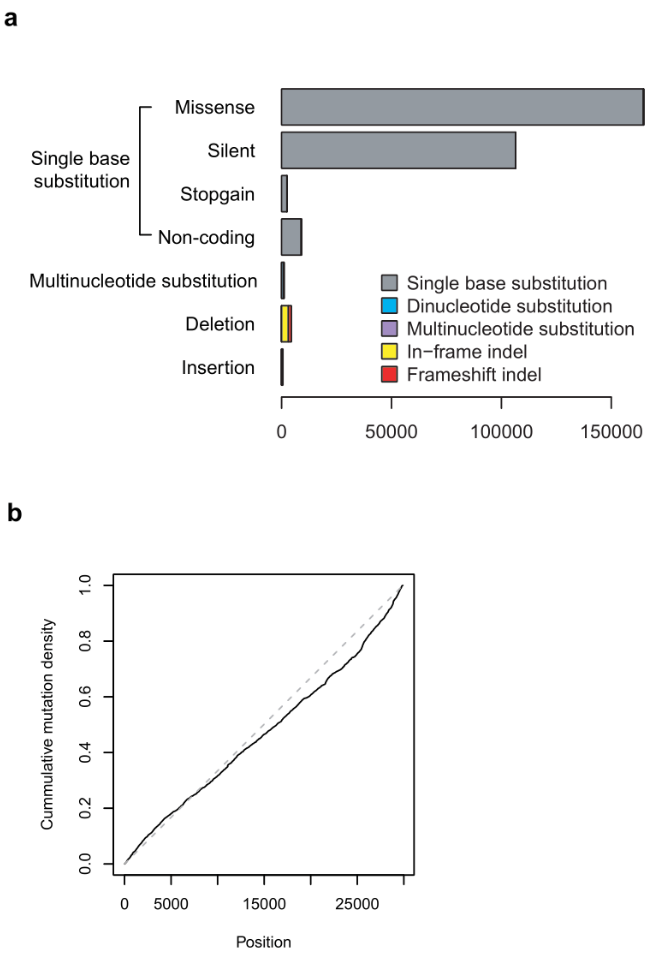

Supplementary Fig. 3: Summaries of SARS-CoV-2 mutations.

a. The plot shows the absolute count of mutations for each of the seven categories: Missense, Silent, Stop-gain, Mutinucleotide substitution, Deletion, and Insertion.

b. Cumulative mutation density along with the positions in the SARS-CoV-2 genome.

Supplementary Fig. 4

a

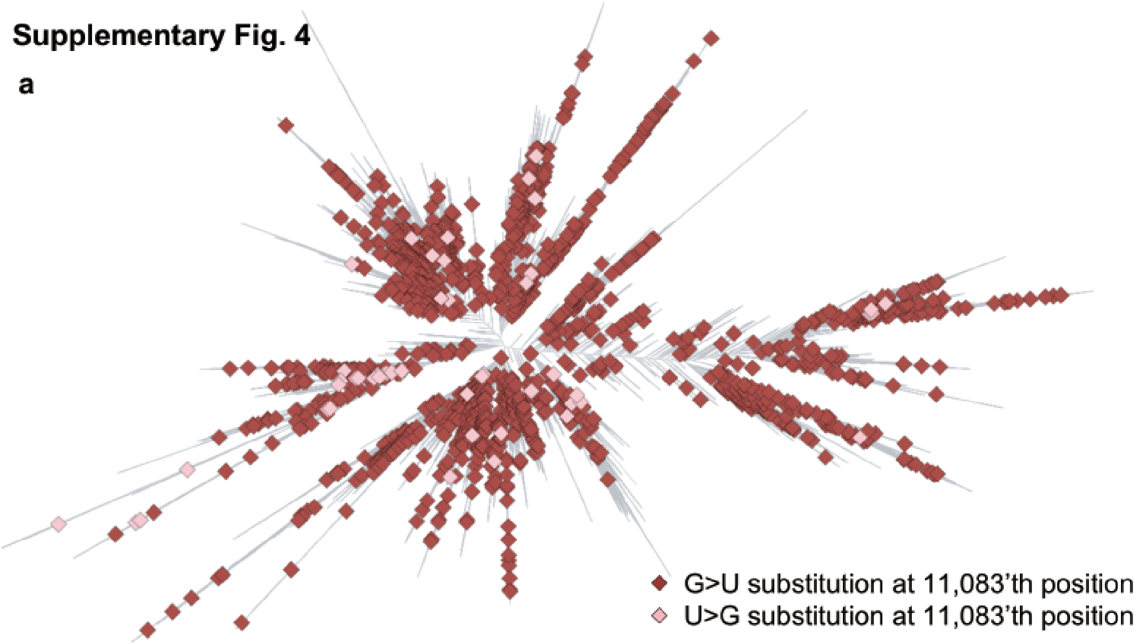

b

COVID-19 Genomics UK (COG-UK) consortium
NextSeq 550, single end viral amplicon sequencing
COG-UK/PHWC-27425

COG-UK/PHEC-18C3E

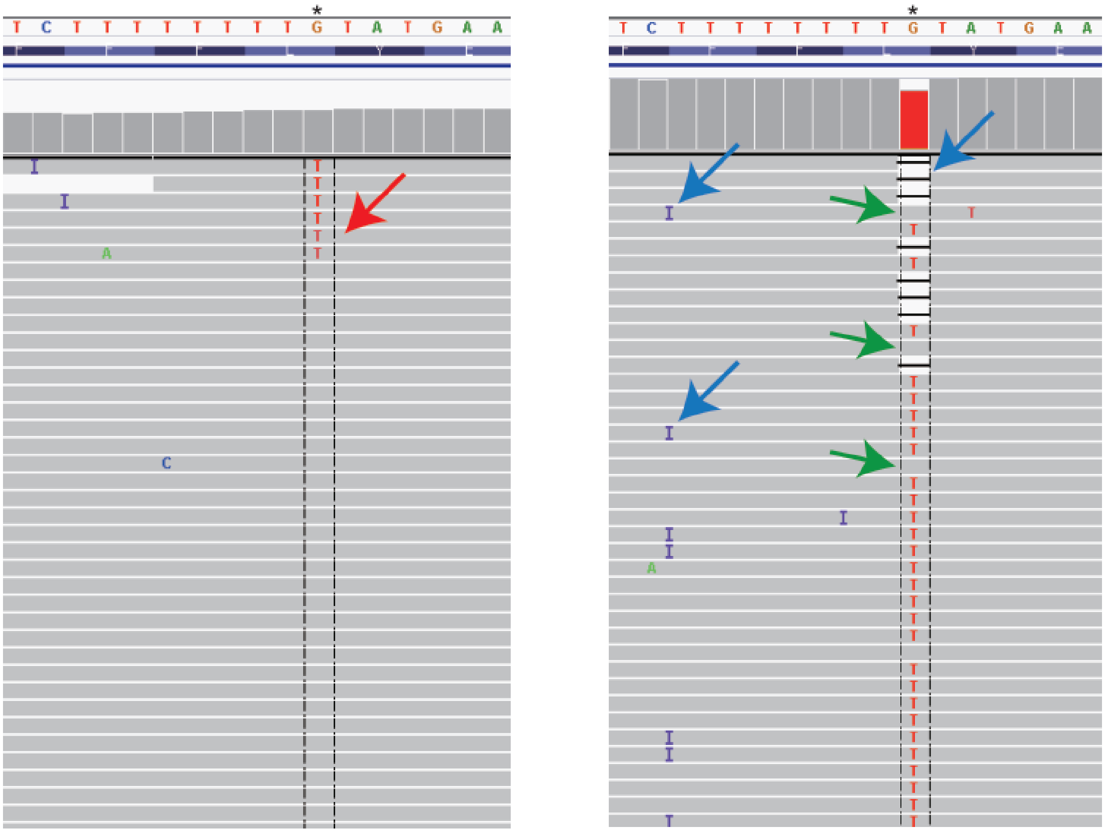

Supplementary Fig. 4: *ORF1ab* L3606F mutation (base position 11,083) located in a homopolymeric sequence.

a. G11083U (or U11083G) mutations occurred 1554 times independently on the phylogenetic tree of SARS-CoV-2 (1505 occurrences of G→U and 49 occurrences of U→G).–

b. Two Illumina short-read sequencing examples at position 11,083 in the SARS-CoV-2 genome. The two samples (COG-UK/PHWC-27425 for left and COG-UK/PHEC-18C3E for right panel) were obtained from the COVID-19 Genomics UK consortium database. On the left side, the position is genotyped as G, and T occurs only in <0.5% reads (60 out of 12289x). Also, a drop in the base quality is shown as a faint base color (red arrow). On the right panel, the site is genotyped as U and often shows aligned reads with inconsistent lengths of U-stretch homopolymer (insertion and deletions are marked with blue arrows) as well as occasional guanine bases. There are 285 reads with a guanine base in that position among 7,326x coverage (haplotype frequency = 4%).

Supplementary Fig. 5

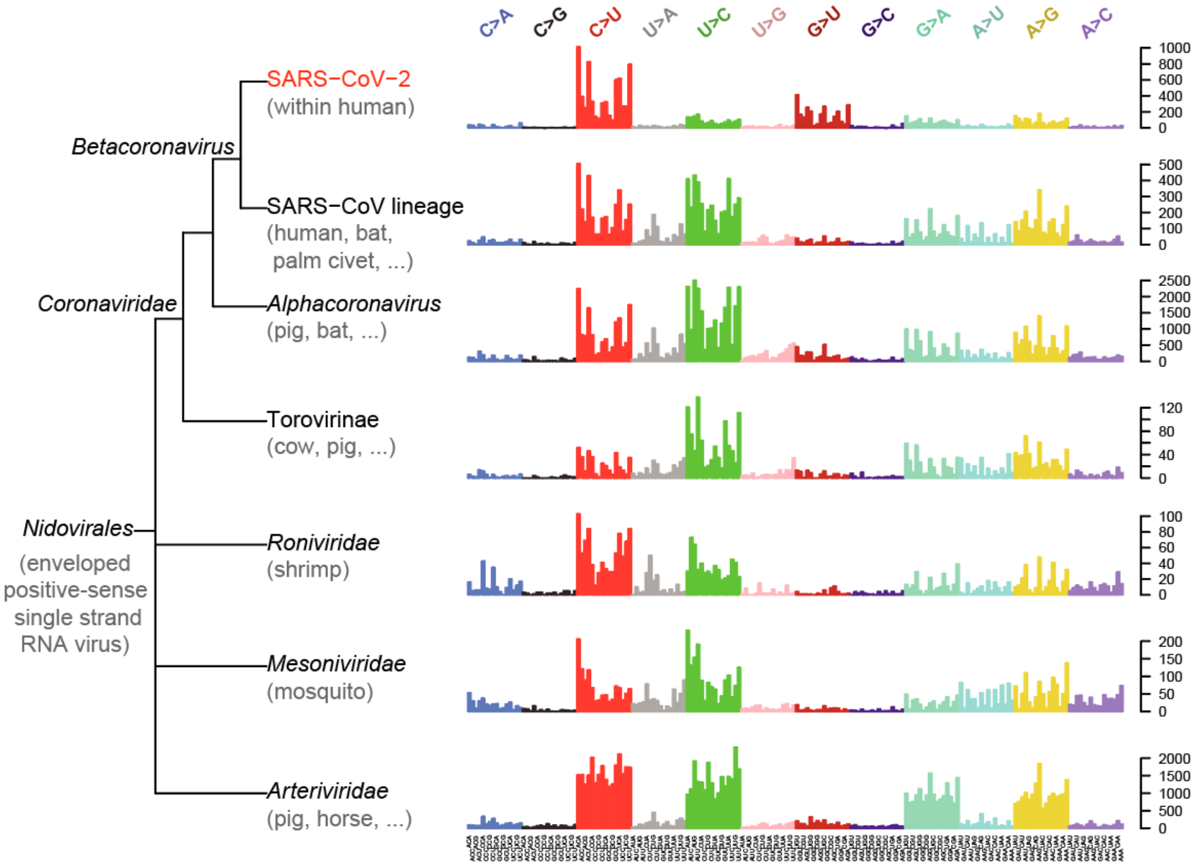

Supplementary Fig. 5: Landscape of mutational signatures in viral speciation.
Spectra of mutational signatures were observed during SARS-CoV-2 spreading in the human population (top panel) and during virus speciation (remaining panels). Except for SARS-CoV-2 and Torovirinae, mutational spectra are largely balanced (C→U ≈ U→C and G→A ≈ A→G).

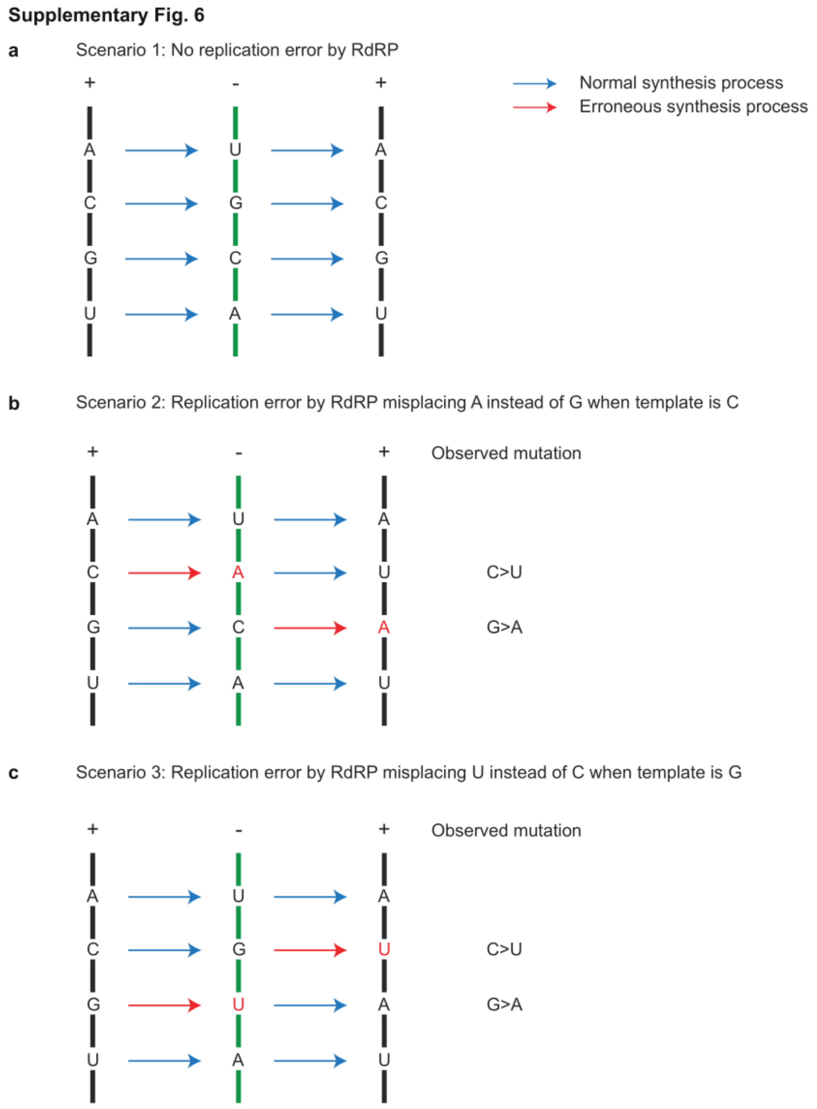

Supplementary Fig. 6: Illustration of potential replication error made by RNA-dependent RNA polymerase (RdRP) in the viral genome.

a. Scenario of no replication error. All Nidovirales, including SARS-CoV-2, invade cells with a single positive-sense RNA strand (the first black vertical bar with marked bases). Using its RdRP, it then copies the genomic RNA into a negative-sense RNA (green vertical bar in the middle). Finally, a positive-sense RNA (the last black vertical bar) is synthesized using the negative-sense RNA as a template.

b and c. Scenarios of replication error resulting in dominant C→U mutations. If C→U dominance is mainly caused by RdRP activity, two major error mechanisms would be either misplacement of A instead of G when the template base is C (**b**) or misplacement of U instead of C when the template base is G (**c**). As the same number of replications with strand switching are expected, in the end, a similar number of G→A mutations to C→U (the symmetric counterpart of G→A by Watson-Crick's base pairing rule) would be observed.

Supplementary Fig. 7

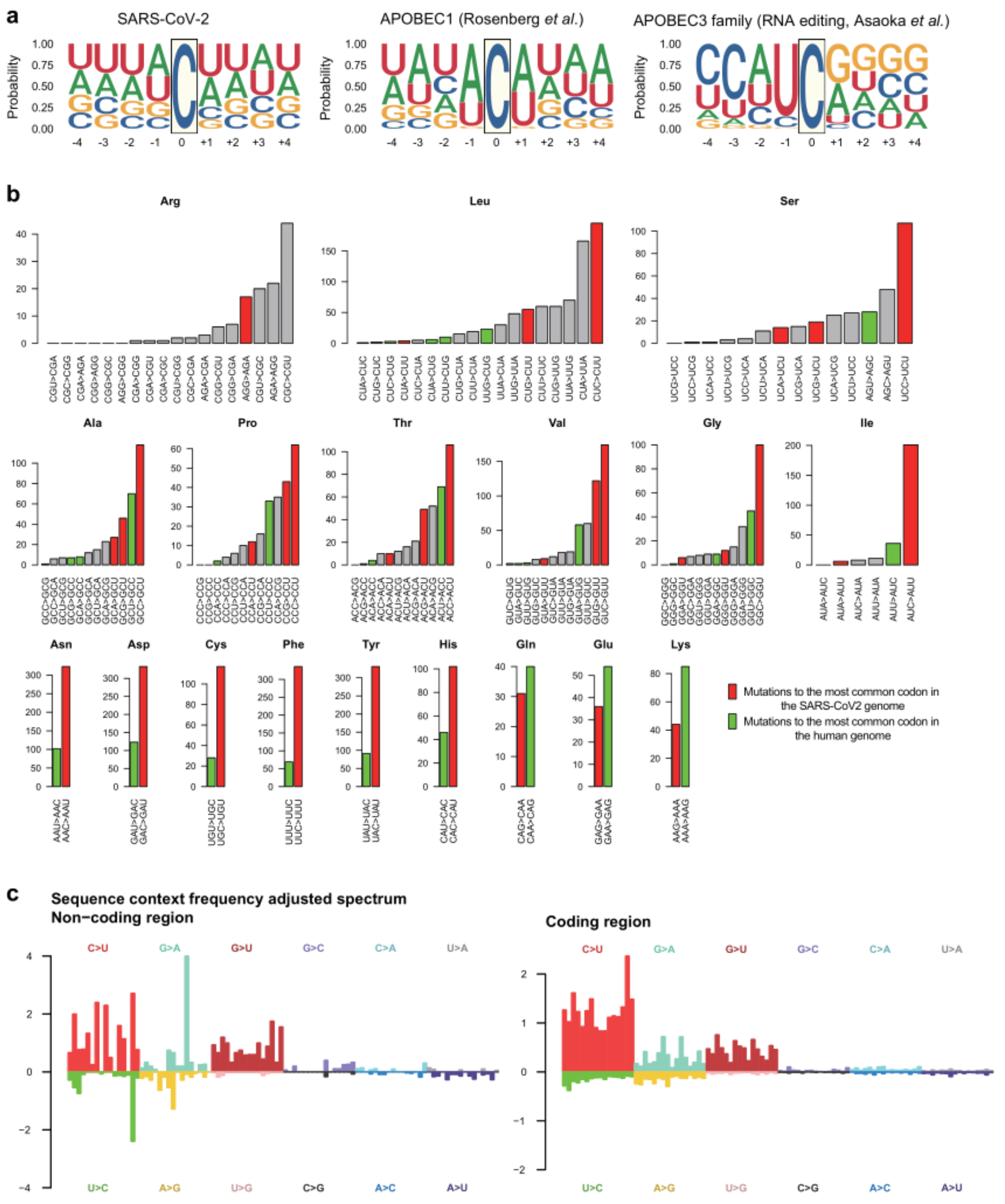

Supplementary Fig. 7: Basis for characterizing the mutational spectrum of SARS-CoV-2: RNA editing, translational selection, and comparison between coding and non-coding regions.

a. The sequence context of a C→U transition (four upstream and four downstream bases) in SARS-CoV-2 (left) and editing sites targeted by APOBEC1 (middle) and APOBEC3 family enzymes (right). The frequency patterns of APOBEC1 and APOBEC3 family enzyme sites are adopted from previous studies[38,79].

b. The mutations are occurring in the direction of strengthening the virus's codon usage bias. These plots show the frequency of codon changes in synonymous mutations by each amino acid. Red color means the mutation change results in the most prevalent codon in the SARS-CoV-2 genome among the synonymous codons. Green means mutations toward the most frequently used codon in the human genome.

c. Comparison of mutational signatures in the mutations of the non-coding and coding regions, adjusted by the frequency of trinucleotide context in the sequences.

Supplementary Fig. 8

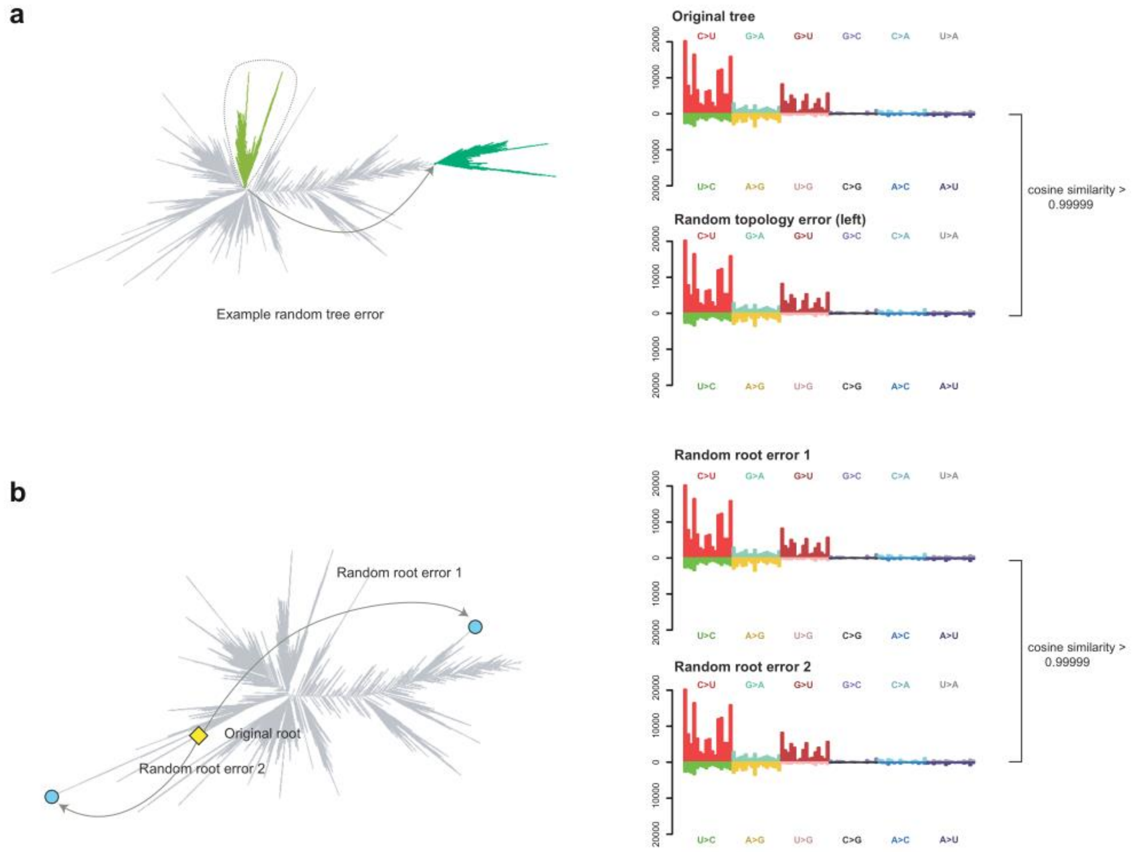

Supplementary Fig. 8: Robustness of the SARS-CoV-2 mutational spectrum.

a. A potential error in tree topology results in very subtle changes in the mutational spectrum.

b. The mutational spectrum is resistant to the mislocation of roots in the phylogenetic tree.

Supplementary Fig. 9

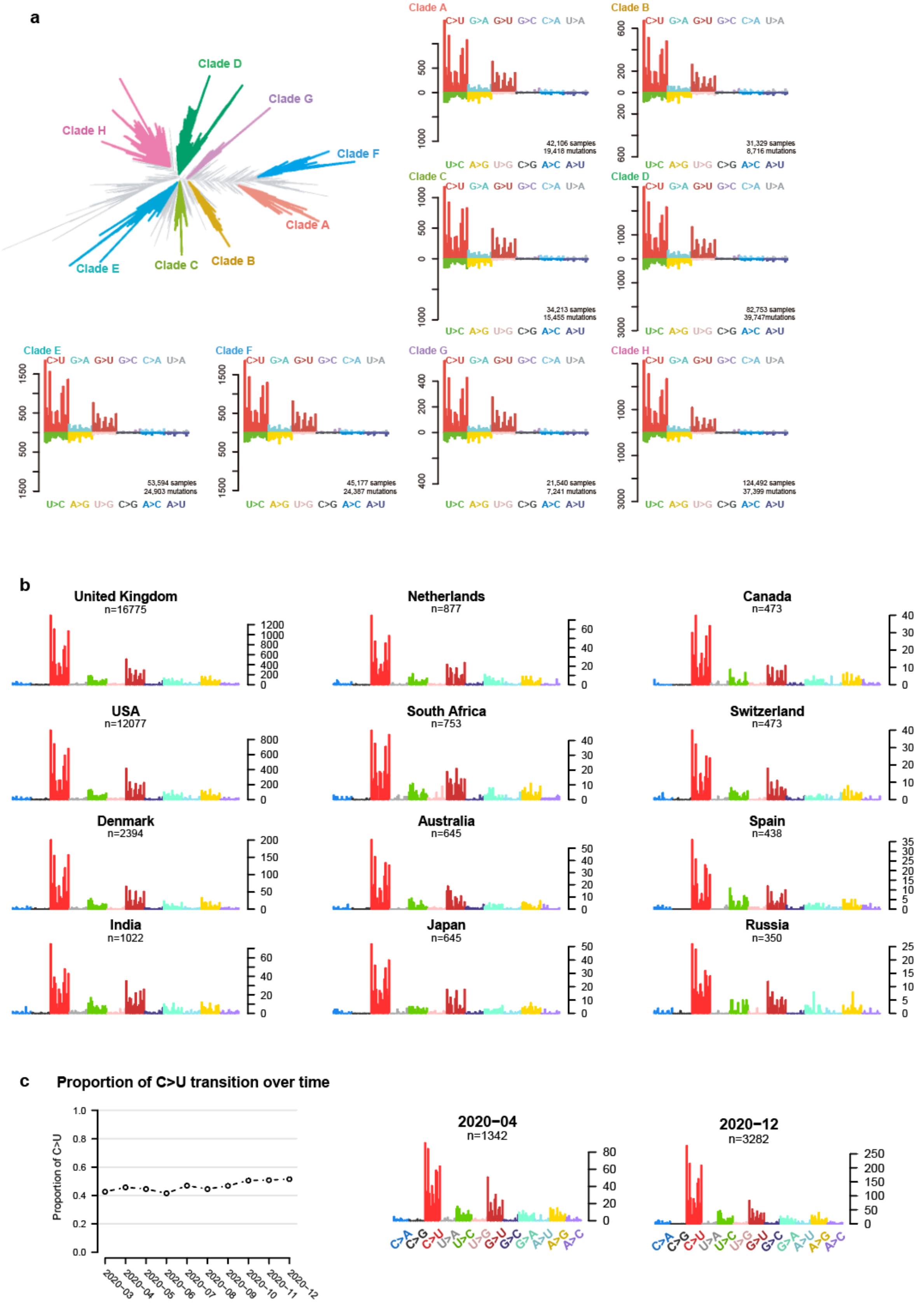

Supplementary Fig. 9: Conserved SARS-CoV-2 mutational spectrum across times and regions

a. Mutational spectra of a few randomly selected sub-clades. The spectrum is highly homogeneous (consistent) across the sub-clades. The order of the peaks is the same as in **Fig. 1b**.

b. Mutational spectra of SARS-CoV-2 by country. Each mutational spectrum constructed with mutations presumably occurred in the country consistent with those mutations in terminal clades, and whose descendants are exclusively shared among patients from the same country. This process was performed within six steps from the most far leaves and excluding terminal edges. The order of the peaks is the same as in **Supplementary Fig. 5**.

c. Proportions of C>U transition and mutational patterns along with the sample collection time. The other months also showed similar mutational spectra (data not shown).

Supplementary Fig. 10

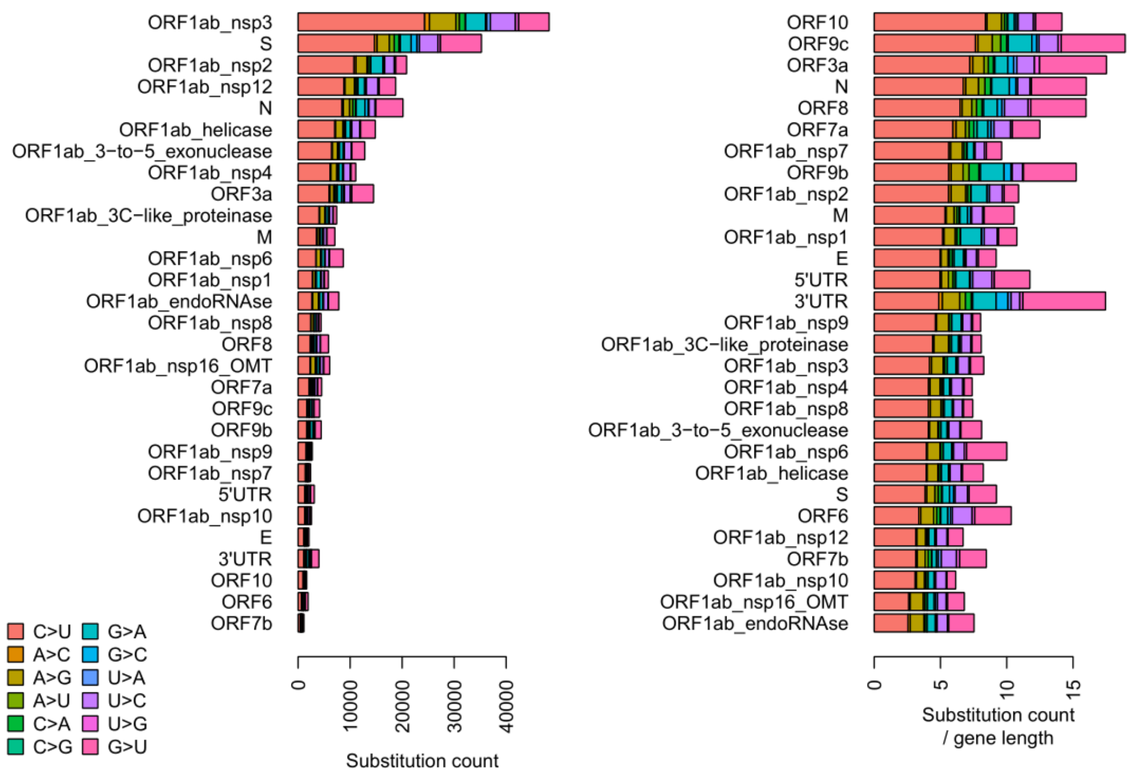

Supplementary Fig. 10: Nonsynonymous substitution counts and length-normalized counts per gene in the SARS-CoV-2 genome.

The genes are arranged in the order of the amount of C>U substitution.
